# Inhibition of MicroRNA 6937 Delays Photoreceptor and Vision Loss in a Mouse Model of Retinitis Pigmentosa

**DOI:** 10.3390/pharmaceutics12100913

**Published:** 2020-09-24

**Authors:** Ander Anasagasti, Araceli Lara-López, Santiago Milla-Navarro, Leire Escudero-Arrarás, María Rodríguez-Hidalgo, Nerea Zabaleta, Gloria González Aseguinolaza, Pedro de la Villa, Javier Ruiz-Ederra

**Affiliations:** 1 Sensorial Neurodegeneration Group, Biodonostia Health Research Institute, 20014 San Sebastian, Spain; anasagasti.85@gmail.com (A.A.); araceli.lara@biodonostia.org (A.L.-L.); leire.escudero@biodonostia.org (L.E.-A.); maria.rodriguezhidalgo@biodonostia.org (M.R.-H.); 2 Viralgen Vector Core, 20009 San Sebastián, Spain; 3 Visual Neurophysiology, IRYCIS, University of Alcala, 28801 Madrid, Spain; santiago.milla@edu.uah.es (S.M.-N.); pedro.villa@uah.es (P.d.l.V.); 4 Gene Therapy and Regulation of Gene Expression Program, CIMA, FIMA, University of Navarra, Navarra Institute for Health Research (IdisNA), 31008 Pamplona, Spain; nzabaleta@unav.es (N.Z.); ggasegui@unav.es (G.G.A.); 5 RETICS OFTARED, 28040 Madrid, Spain

**Keywords:** microRNA, miRNA, miR, retina, retinal disease, IRD, RP, photoreceptor, miRNA-mRNA interaction, rd10, mouse models, whole transcriptome

## Abstract

Inherited retinal dystrophies (IRDs) are a group of rare retinal conditions, including retinitis pigmentosa (RP), caused by monogenic mutations in 1 out of more than 250 genes. Despite recent advancements in gene therapy, there is still a lack of an effective treatment for this group of retinal conditions. MicroRNAs (miRNAs) are a class of highly conserved small non-coding RNAs that inhibit gene expression. Control of miRNAs-mediated protein expression has been described as a widely used mechanism for post-transcriptional regulation in many physiological and pathological processes in different organs, including the retina. Our main purpose was to test the hypothesis that modulation of a group of miRNAs can protect photoreceptor cells from death in the rd10 mouse model of retinitis pigmentosa. For this, we incorporated modulators of three miRNAs in adeno-associated viruses (AAVs), which were administered through sub-retinal injections. The results obtained indicate that inhibition of the miR-6937-5p slows down the visual deterioration of rd10 mice, reflected by an increased electroretinogram (ERG) wave response under scotopic conditions and significant preservation of the outer nuclear layer thickness. This work contributes to broadening our knowledge on the molecular mechanisms underlying retinitis pigmentosa and supports the development of novel therapeutic approaches for RP based on miRNA modulation.

## 1. Introduction

Inherited retinal dystrophies (IRDs) are a group of rare, heterogeneous eye disorders caused by mutations in more than 250 genes, leading to progressive photoreceptor death and vision loss [1]. Retinal degenerations include multifactorial diseases such as Leber congenital amaurosis, age-related macular degeneration, Stargardt disease, and retinitis pigmentosa (RP) [2,3,4].

Despite recent advancements in therapeutic approaches for inherited retinal diseases, there is still a lack of an effective treatment for the main types of IRDs. One of the most advanced therapeutic options is gene therapy, with a treatment currently available for an aggressive form of RP, Leber Congenital Amaurosis, caused by mutations in the RPE65 gene [5,6]. However, to develop treatments for IRDs caused by mutations in all IRDs related-genes, complementary therapeutic options to gene therapy would be much welcomed.

MicroRNAs (miRNAs or miRs) are a class of highly conserved ~22 nucleotide long non-coding RNAs that have a repressive impact on gene expression in a sequence-specific manner [7]. Control of protein expression by miRNAs has been described as a widely used mechanism in a large number of both physiological and pathological processes, including immune response and oncogenesis or cell proliferation, differentiation, and death, among others [8,9]. Therefore, there is growing evidence of the participation of miRNA’s—and other untranslated transcripts, such as long-non coding RNAs’—dysregulation in a broad spectrum of health problems, including heart and inflammatory diseases and various types of cancer and hereditary diseases such as cystic fibrosis [7,10,11,12,13,14,15,16,17,18,19,20].

Concerning the retina, a growing list of differentially expressed miRNAs has been described to be involved in retinal diseases, in both humans and animal models [21]. This includes miR-146a and miR-195 in diabetic retinopathy [22,23]; miR-9, miR-34a, miR-125b, and miR-155 in macular degeneration [24,25]; and miR-125a and miR-17 in retinoblastoma [6,26]. In RP, a common pattern of aberrantly expressed miRNAs, miR-1, miR-133, and miR-142, was observed in four murine models of the disease, involving different genes (rho and rds) and inheritance patterns [27,28]. More recently, using an in vitro model of RP, Donato and colleagues found 23 grouped miRNAs with altered expression in human RPE cells under oxidative stress conditions [29]. Additionally, we have recently found a group of differentially expressed miRNAs in the widely accepted rd10 mouse model of RP [30].

miRNA mimics and miRNA inhibitors currently in preclinical development have shown promise as novel therapeutic agents. Multiple technological platforms have been developed for miRNA isolation, quantitation, profiling, and target detection, as well as to modulate their expression (inhibition/upregulation) in vitro and in vivo [31,32,33,34]. Adeno-associated virus (AAV)-based recombinant vectors can be used for the in vivo delivery of miRNA modulatory sequences to different organs or tissues, including the retina.

The main goal of the present work was to test the hypothesis that modulation of a group of miRNAs by incorporating their modulatory sequences in AAVs-based vectors might represent an attractive strategy to protect photoreceptor cells from death in the rd10 mouse model.

## 2. Materials and Methods

### 2.1. Ethics Statement and Animal Handling

Animal handling and experiments were conducted following the Association of Research in Vision and Ophthalmology (ARVO) Statement for the Use of Animals in Ophthalmic and Vision Research, and were approved by the Animal Care and Use Committee of Donostia University Hospital and the Clinical Research Ethics Committee of the Basque Country, Spain (CEEA16/013, 13 January 2017)). We used age-matched mice C57BL/6J (wild-type, WT) as controls, and a congenic inbred strain of Pde6b^rd10^ (rd10) mice (Jackson Laboratory, Bar Harbor, ME, USA). Animals were maintained under a 12 h light/12 h dark cycle at 22 °C, with controlled humidity (45–55%) and with water and food provided ad libitum, at the Animal Facility of BioDonostia Health Research Institute in San Sebastian (Spain).

### 2.2. Sample Collection

Rd10 (*n* = 45) and age-matched WT (*n* = 10) mice were euthanized by cervical dislocation and CO_2_ inhalation. To minimize circadian effects on miRNA expression, mice were euthanized at the same time interval.

Retinal tissue. Once isolated, retinas were kept at 4 °C until their use. Total RNA was isolated with the miRNeasy Mini Kit (Qiagen, Woburn, MA, USA) following the manufacturer’s instructions and kept at −80 °C until its use. RNA samples with A260/A280 > 1.8 were used.

### 2.3. Generation of Adeno-Associated Viruses (AAVs) Expressing miRNA Modulators

Three miRNA modulating plasmids were acquired from GeneCopoeia (Rockville, MD, USA): two inhibitors for miR-6240 and miR-6937-5p (miArrest™ miRNA Inhibitors), and a precursor for miR-142a-5p (miExpress™ miRNA Expression Precursor). Controls consisted of plasmids containing an artificial nucleotide sequence with no complementarity to any known mRNA in the genome (miR scramble). All plasmids expressed enhanced green fluorescent protein (eGFP, miRNA precursors) or mCherry (miRNA inhibitors).

After plasmid transformation into competent bacteria (*E. coli* XL1), amplification, and purification (Plasmid DNA Purification Kit, Qiagen, Hilden, Germany), the expression cassettes containing the miRNA modulatory sequences and the reporter gene were isolated and cloned into the AAV genome plasmid using standard molecular biology techniques to generate the plasmid shown in Figure S1.

In all the constructs, the expression cassette was flanked by AAV2 WT inverted terminal repeats (ITRs). Then, recombinant AAV Anc80L65 (AAV-Anc80) vectors were produced by polyethyleneimine-mediated cotransfection in HEK-293T cells of the following plasmids: the plasmid containing the AAV genome (Figure S1) pKan-Anc80AAP-L0065 (kindly provided by Dr. Luk H. Vandenberghe) and an adenoviral helper plasmid (pDF6) were used, as previously described [35]. After Iodixanol gradient purification, the different vectors were tittered by real time quantitative PCR, obtaining the following titers: vectors used were as follows: ssAAV-Anc80-anti-miR-6937-5p, ssAAV-Anc80-anti-miR-6240, ssAAV-Anc80-scramble-inhibitor, ssAAV-Anc80-precursor-miR-142a-5p, and ssAAV-Anc80-scramble-precursor.

### 2.4. Sub-Retinal Injections

Sub-retinal (SR) injections were performed in a total of 55 mice (110 eyes), in 10 WT and 45 rd10 mice at postnatal day 10 (P10) with no gender distinction. At least 15 mice were used for each miRNA modulator (miR-6937-5p, miR-6240, and miR-142a-5p). The AAV-Anc80 vector carrying the miRNA modulator sequence was injected into the right eye, and ssAAV-Anc80 carrying the scramble control sequence was injected into the left eye (control eye of the experimental procedure). Mice were anesthetized subcutaneously with ketamine solution (70 mg/Kg) and xylazine (7 mg/Kg), and topically by ocular instillation of 0.5% oxybuprocaine. Mydriasis was induced by ocular instillation of 1% tropicamide (Alcon Cusí S.A., El Masnou, Barcelona, Spain). Sub-retinal injections were performed consecutively in both eyes, using a 10 μL WPI syringe (NanoFil Syringe, WPI, Sarasota, FL, USA). First, an incision was made at the scleral limb using a 34G beveled tip needle, after which a 33G blunt tip needle was introduced through the generated incision directed into the sub-retinal cavity. Then, 1 μL of AAV solution was injected into each eye, inducing a transitory retinal detachment.

### 2.5. Electroretinogram Recordings

We used the procedure reported previously with minor modifications [36]. Briefly, after overnight dark adaptation, electroretinogram (ERG) recordings were performed in dim red light at P17 and P25, which we considered sufficient time for the photoreceptor cells to be transduced by the ssAAV-Anc80 vectors. Mice were anesthetized with intraperitoneal administration of a saline solution containing ketamine (95 mg/kg) and xylazine (5 mg/kg), and kept on a 37 °C heating pad. Ocular instillation of 1% tropicamide was applied to induce mydriasis. ERG responses were recorded from both eyes in response to a Ganzfeld stimulator under light flashes. A total of 4 to 64 consecutive stimuli were averaged, with an interval between light flashes in scotopic conditions of 10 s for dim flashes and of up to 60 s for the highest intensity for every light intensity used. For photopic conditions, we used a one-second interval between light flashes. ERG signals were amplified, and band filtered between 0.3 and 1000 Hz (CP511 AC amplifier; Grass Instruments, Quincy, MA, USA). Electrical signals were digitized at 20 kHz with a power laboratory data acquisition board (AD Instruments, Chalgrove, UK). ERGs were performed using an electrode fixed to a corneal lens (Burian-Allen electrode; Hansen Ophthalmic Development Laboratory, Coralville, IA, USA) and a reference electrode fixed to the mouth, with a ground electrode placed in the tail. Rod-mediated responses were recorded from light flashes ranging from −4 to −1.5 log cd s/m^2^ with dark-adapted mice. Mixed rod- and cone-mediated signals were recorded in response to −1.5 to 1.5 log cd s/m^2^ light flashes. Oscillatory potential (OP) was isolated using 0.48 log cd s/m^2^ white flashes, with a recording frequency ranging between 100 and 10,000 Hz. For recording of cone-mediated responses, we applied light flashes ranging from 0.5 to 2 log cd s/m^2^ on a rod-saturating background of 30 cd/m^2^. Flicker responses (20 Hz) to light flashes of 1.5 log cd s/m^2^ were also recorded on a rod-saturating background. Amplitudes of the a-wave and b-wave were averaged, using data provided by a researcher with no information regarding the experimental condition of mice.

### 2.6. Histological Evaluation

Enucleated eyes were fixed in 4% paraformaldehyde in PBS, cryoprotected in 30% sucrose in PBS, embedded in OCT (Tissue Tek, Sakura Finetek, Tokyo, Japan), and cryosectioned at 7 mm. Cell nuclei were revealed with DAPI staining and analyzed with a Nikon Eclipse 80i microscope (Nikon, Tokyo, Japan) coupled to a Nikon DS-U2 digital camera (Nikon, Tokyo, Japan). A total of 30 eyes were analyzed, with 6 per treatment (miRNA modulators and scramble). Four different sections per eye globe and images from five areas/sections were analyzed (central, mid-peripheral, and peripheral retina). The thickness of the inner nuclear layer (INL) and the number of photoreceptor rows were analyzed using ImageJ (NIH-Image, 1,38x, Bethesda, MD, USA).

### 2.7. Clariom S Arrays-Based Transcriptome Analysis

The whole transcriptome of three retinas from three rd10 mice treated at P10 with AAV-Anc80-anti-miR-6937-5p and their three contralateral retinas treated with AAV-Anc80-scramble-inhibitor were analyzed at P22, using six Clariom S PICO arrays (Affymetrix, Santa Clara, CA, USA). To assess a possible effect of SR injections on mRNA expression levels, the retinas from three eyes that received no SR injection were used as controls.

Raw data were analyzed using the Transcriptome Analysis Console (TAC) Software (Affymetrix, 4.0.1, Santa Clara, CA, USA). Only those genes that showed, concomitantly, (1) differential expression levels between treated and control groups (scramble and injection controls) and (2) no differences between scramble and injection controls, were considered for further analysis. The selected genes were subjected to genetic ontology (GO), biological pathway enrichment analysis, and miRNA-mRNA interaction network studies, following the same procedure detailed in [30]. We analyzed the relative miRNA expression within photoreceptor cells compared to the rest of the retina in a selection of seven miRNAs (Figure S2). For this, we used RT-qPCR (miScript Primer Assays and miScript SYBR Green PCR Kit from Qiagen, Hilden, Germany). Each biological sample was amplified in triplicate as previously described [30].

### 2.8. Validation of mRNA Expression by Quantitative PCR

Expression levels of differentially expressed mRNAs were validated by quantitative PCR (RT-qPCR), following previously reported methods with minor modifications [30]. Briefly, RT-qPCR was done using miScript Primer Assays and SYBR Green PCR Master Mix (Applied Biosystems, Foster City, CA, USA). Biological samples were amplified in triplicate. Threshold cycle values for mRNA expression were normalized to the mean expression values of Gapdh, Tubb5, and b-Act genes, based on their stable expression levels across all samples (r^2^ = 0.905 and *p* < 0.001). Information of primer sequences used for mRNA expression levels validation is shown in Table S1.

### 2.9. miR-6937-5p In Vitro Modulation

In order to simulate the increased expression of miR-6937-5p observed in the retinas of rd10 mice, we used the MU-PH1 cell line kindly provided by Professor Nicolas Cuenca at the University of Murcia, Spain. This cell line is derived from Müller cell precursors from adult mouse retinas, with photoreceptor, glial cell, and stem cell characteristics [37]. Cells were seeded in 96-well plates with 250 μL of DMEM/F12 medium with 2% FBS and 1% antibiotic (penicillin/streptomycin 10.000 μg/mL), supplemented with 2% B27 complement and 20 ng/mL human FGF-2 factor at 37 °C and 5% CO_2_/95% O_2_. Cells were seeded at ≈ 2 × 10^4^ cells/well. After 24 h, cells were transfected with miRIDIAN mmu-miR-6937-5p mimic (Dharmacon, Lafayette, CO, USA) and labeled with Dy547, with the aid of Lipofectamine RNAiMAX (Invitrogen, Waltham, MA, USA), following the manufacturer’s instructions. Transfection yield was determined by flow cytometry (Guava easyCyte 8HT, Millipore, MA, USA), based on Dy547 emission detection. Increased levels of miR-6937-5p were measured by RT-qPCR using miScript Primer Assays and the miScript SYBR Green PCR Kit.

### 2.10. miR-6937-5p In Vitro Induced Cell Viability and Cytotoxicity

We used the non-radioactive cytotoxicity assay CytoTox 96 (Promega, Madison, WI, USA) following indications [38].

### 2.11. Statistical Analysis

Statistical significance was analyzed using the Student’s *t*-test or Mann–Whitney test for normal or non-parametric distributions, respectively, using SPSS Statistics v22 software (IBM, Armonk, NY, USA). Measures with a probability of less than 0.05, with Bonferroni-adjusted *p*-values, were considered statistically significant differences.

## 3. Results

**Figure 1:**
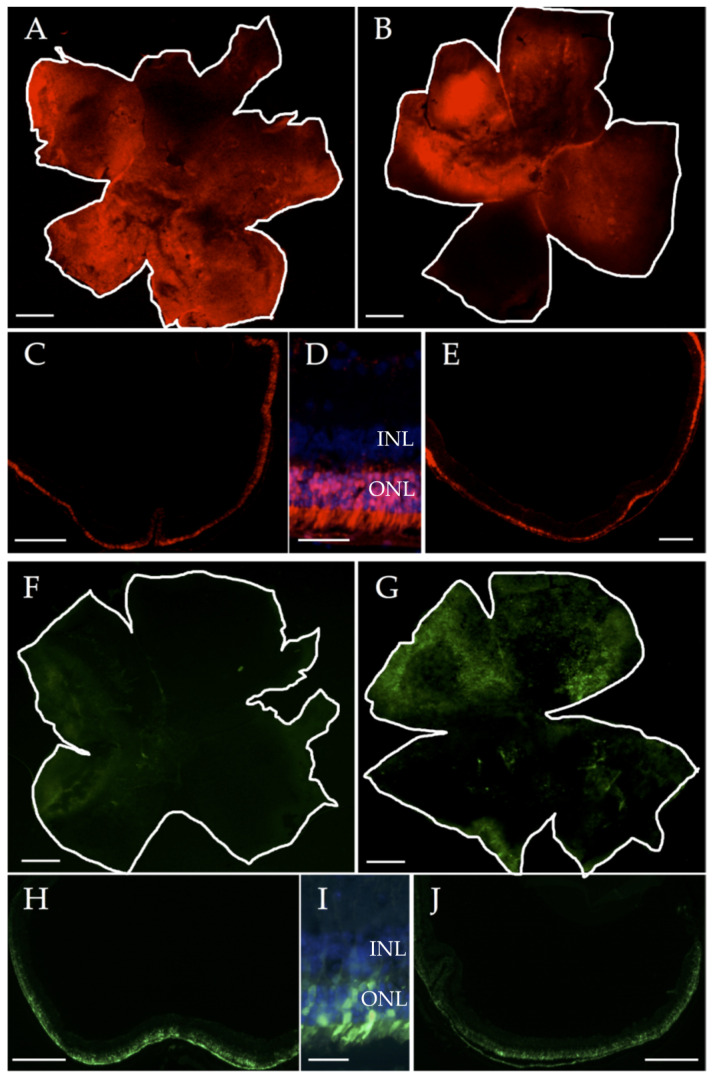
Representative images of mCherry (**A**–**E**, red) and GFP (**F**–**J**, green) reporter expression after sub-retinal injections of ssAAV-Anc80 solutions, as seen in the whole mount and retinal sections. (**A**,**C**) Retinas treated with ssAAV-Anc80-anti-miR-6937-5p. (**B**,**E**) Retinas treated with ssAAV-Anc80-scramble-inhibitor. (**F**,**H**) Retinas treated with ssAAV-Anc80-pre-miR-142a-5p. (**G**,**J**) Retinas treated with ssAAV-Anc80-scramble-precursor. (**D**,**I**) Expression of AAV-Anc80 is restricted to photoreceptor cells, as can be observed in high magnification of retinal sections with nuclei stained with DAPI (blue). Abbreviations: INL: inner nuclear layer; ONL: outer nuclear layer; AAV: adeno-associated virus. Scale bars: 400 μm, except in (**D**,**J**): 50 μm.

Prior to miRNA modulation in rd10 mice, we performed in vivo tests in WT mice to verify the generated AAVs’ ability to transduce retinal photoreceptor cells. For this, we performed sub-retinal injections in 10 C57BL/6J WT mice, with two mice (four eyes) for each of the five AAV-Anc80 generated. We verified that both AAVs carrying miRNA inhibitory sequences and miRNA precursors could transduce photoreceptor cells selectively, as shown in Figure 1.

However, we detected differences in the transduction capacity among different types of AAVs employed. In general, AAVs with miRNA expression inhibitory sequences transduced photoreceptors more efficiently than AAVs carrying miRNA precursor sequences, with an average of 50–60% of the retina transduced with inhibitors-expressing AAVs, and with only 20–30% of the retina transduced with precursors-expressing AAVs (Figure 1 and Figure S3).

### 3.1. miR-6937-5p Inhibition Slows Down Visual Loss in rd10 Mice

We assessed the preservation of visual function by electroretinography recordings from rd10 mice to test the effect of modulating the expression of miR-6240, miR-6937-5p, and miR-142a-5p on visual response. Mice received the AAVs carrying miRNAs modulatory sequences on their right eyes. Scramble AAVs were administered to left eyes as negative controls. For each treatment, 15 mice were analyzed at both P17 and P25 in the dark- and light-adapted conditions. ERG-wave amplitudes were determined.

**Figure 2:**
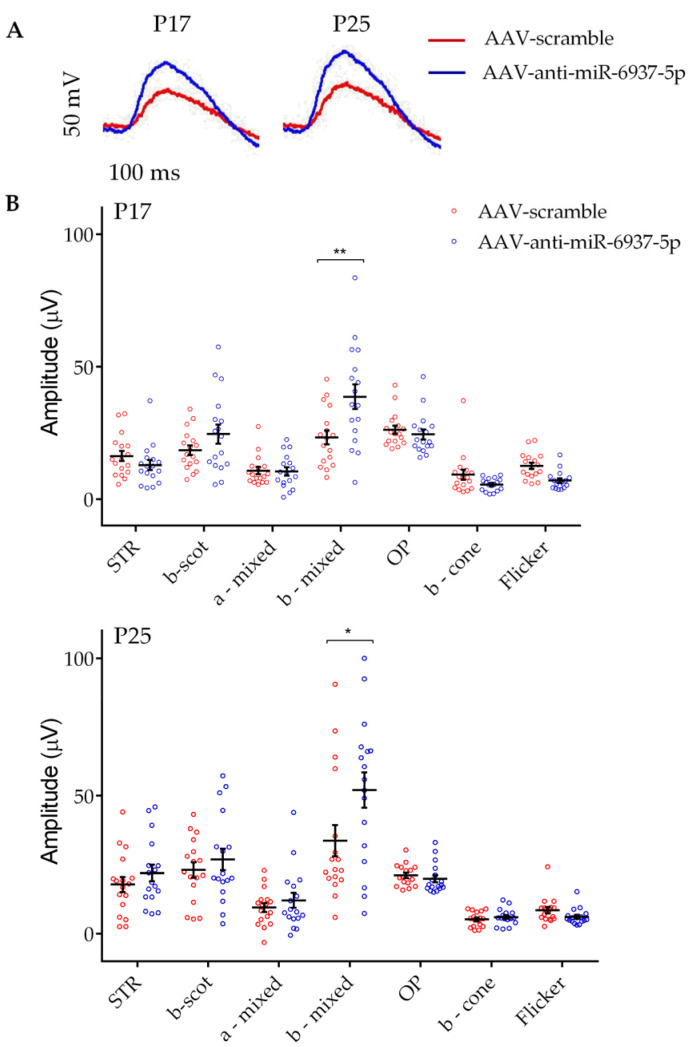
Increased amplitude of visual response in eyes treated with miR-6937-5p inhibitors. A. Representative electroretinogram (ERG) recordings of rd10 mice treated with miR-6937-5p inhibitor (blue) solution and eyes treated with scramble solution (red). (**A**) A significantly increased mixed response under scotopic conditions is shown, at both P17 and P25. (**B**) Representative scatter plots of different ERG parameters analyzed at both P17 and P25. *N* = 15 mice per treatment. Abbreviations: STR: scotopic threshold response; scot: scotopic; OP: oscillatory potential. Data are presented as mean values ± S.E.M. * *p* < 0.05, ** *p* < 0.01 (Student’s *t*-test).

As a result, we observed significant differences in the averaged ERG amplitudes in b-mixed waves under scotopic conditions in ERG from eyes injected with miR-6937-5p inhibitor, compared with ERG recorded in contralateral scramble eyes. The mixed response is the combination of a wave (initial downward response) that corresponds to the activity of cones and rods, followed by the upward response of bipolar cells post-synaptic to photoreceptors (b-wave). This increase in amplitude was significant at both P17 (38.7 ± 4.63 vs. 23.32 ± 2.65 μV) and P25 (52.06 ± 6.46 vs. 36.6 ± 5.72 μV) (*p* < 0.05) (Figure 2). No significant differences were observed in the rest of the ERG parameters analyzed.

In contrast, mice treated with miR-6240 and miR-142a-5p modulators showed no significant differences between treated and control eyes in any of the ERG parameters analyzed, including the b-wave mixed response (Figure S4).

### 3.2. miR-6937-5p Inhibition Delays Photoreceptor Cell Loss in rd10 Mice

**Figure 3:**
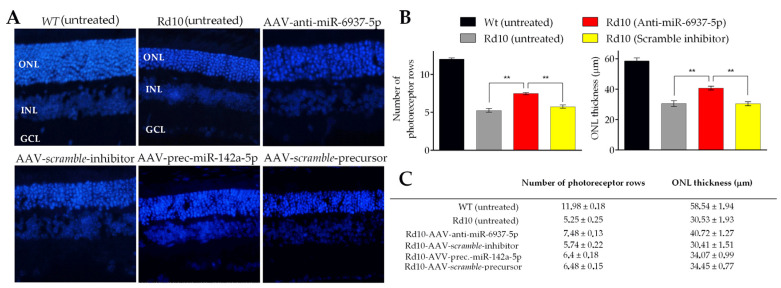
Histological analysis of rd10 mice retinas treated with miRNA modulators. (**A**) A significantly increased ONL (nuclei stained with DAPI) is observed in the retinas of rd10 mice treated with mir-6937-5p inhibitors, whereas no differences are observed in the rest of the conditions analyzed. Sections of untreated retinas from wild-type and rd10 mice are shown for comparative purposes. (**B**) Histograms show the mean values of the number of photoreceptor nuclei rows (left) and outer nuclear layer (ONL) thickness (right) from the retinas of rd10 mice submitted to miR-6937-5p inhibition. We used retinas from rd10 mice injected with scramble miRNA sequences as controls. Values from untreated wild-type (WT) and rd10 mice are also shown. (**C**) Summary of histological parameters analyzed. Abbreviations. GCL: ganglion cell layer; INL: inner nuclear layer; ONL: outer nuclear layer. Data are presented as mean values ± S.E.M. ** *p* < 0.01 (Student’s *t*-test).

To evaluate the potential protective effect of in vivo administration of miRNA modulators, we measured the number of photoreceptor nuclei rows and the thickness of the outer nuclear layer (ONL) in histological retinal sections stained with DAPI at P22 (Figure 3).

As photoreceptor degeneration is not homogeneous throughout the retina [39], the effects of the treatments were evaluated in different regions: from the nasal area to the temporal margins. We found a significant effect in the retina of rd10 mice treated with anti-miR-6937-5p, in terms of both the number of rows of photoreceptor cells and ONL thickness (Figure 3B,C). We found a mean of 7.48 ± 0.13 rows in the retinas treated with anti-miR-6937-5p compared with 5.55 ± 0.23 rows of photoreceptor cells in controls at P22. Regarding retinal thickness, the ONL of retinas treated with miR-6937-5p inhibitor measured an average of 40.72 ± 1.27 μm, compared with 29.42 ± 1.53 μm of control retinas. miR-6937-5p inhibition slowed the rate of degeneration of photoreceptor cells by 28.91% in terms of the number of photoreceptor rows and 36.42% in terms of ONL thickness.

Concerning mice treated with miR-6240 and miR-142a-5p modulators, no differences were detected in any of the two parameters analyzed, consistent with data obtained from ERG recordings (Figure 3C).

### 3.3. In Vivo Inhibition of miR-6937-5p in rd10 Mice Modulates the Expression of 101 Genes

Analysis of the transcriptome using ClariomS arrays (Affymetrix) allowed us to identify a set of 101 mRNAs with increased expression (fold-change >1.5) among mice treated with miR-6937-5p inhibitors. For controls, we used expression data obtained from the retinas of eyes treated with scramble solution and from eyes that received no injections at all. On the basis of gene ontology (GO) studies and biological pathway analysis, we found a set of genes involved in biological pathways that may play important roles in the partial functional photoreceptor rescue detected by ERGs and histological analysis. Among them, we selected those differentially expressed genes involved in anti-apoptotic processes (Bcl2a1d, Mapk1, Itch, Arel1), immune system regulators (Angptl1, Trim21), inflammatory processes (Jak3), transcription regulation (Atxn2l, Tle2, Rps6ka5), or intracellular transport (Klc1, Slc6a9, Cog2), as well as those involved in retinal physiological processes (Cryba1, Gucy2f, Crygc), among others. Because our mouse model carries a mutation in PDE6B, we also selected genes involved in calcium homeostasis or regulation (Atp2c1, Cpne8, Cldn5, Ramp3). Table 1 summarizes the main differentially expressed mRNA and their associated biological pathways, after in vivo modulation of miR-6937-5p.

We next performed a miRNA-mRNA interaction analysis to identify those mRNAs with augmented expression levels that were also predicted targets for miR-6937-5p. We identified three candidates: Angptl1, Bcl2a1d, and Exosc6. Angptl1 is associated with the Akt signaling pathway, promoting survival and growth in response to extracellular stimuli. Bcl2a1d and Exosc6 are associated with negative regulation of apoptosis (Table 2).

### 3.4. Increased Cellular Cytotoxicity Induced by Over-Expression of miR-6937-5p in the MU-PH1 Photoreceptor-Like Cell Line

Finally, to test the effect on cellular viability upon increased expression of miR-6937-5p, we used a photoreceptor-like cell line, MU-PH1, generously provided by Professor Nicolas Cuenca from U. of Alicante, Spain [37]. MU-PH1 cells were transfected with miR-6937-5p mimic using the miRIDIAN system (Dharmacon). After 48 h of incubation with miRIDIAN miR-6937-5p Mimic Transfection, a 174-fold increase in the expression of miR-6937-5p was achieved in 29.9% of MU-PH1 cells, compared with cells treated with miRNA mimic negative control. This increased expression of miR-6937-5p was correlated with increased cytotoxicity of MU-PH1 cells (Figure S5).

## 4. Discussion

On the basis of the advances made by other research groups who have successfully used RNA interference technology for the in vitro and in vivo modulation of miRNA expression [40,41], and on previous observations regarding miRNA and mRNA aberrantly expressed, with relevance to retinitis pigmentosa [30,42,43], we worked under the hypothesis that the modulation of differentially expressed miRNAs in the retina of rd10 mice could slow down the progression of photoreceptor cell death.

For this purpose, we selected 3 miRNAs (miR-142a-5p, miR-6240, and miR-6937-5p) out of 19 candidates we previously identified that may play fundamental roles in the physiopathology and/or in the progression of the retinal degeneration [30]. The selection of these three miRNAs was based on (1) a putative link of their target mRNAs to degenerative processes, concomitant with an inverse relationship in their expression (i.e., up-regulated miRNA, vs. downregulated target mRNA, or vice versa); and (2) high and sustained expression fold-change values. The selection of miR-142a-5p was also supported by previous works that associated this miRNA with the degenerative processes in the retina [27,44].

We chose the viral capsid serotype Anc80 to generate our miRNA modulating AAVs. This serotype is a predecessor of the widely studied AAV capsid serotypes 1, 2, 8, and 9, a potent in vivo gene therapy vector with the ability to target and infect the liver, muscle, and retina very effectively [45]. In particular, AAVAnc80 has demonstrated a high level of retinal pigment epithelium and photoreceptor targeting in both mice and non-human primates [46].

Our results showed that AAVAnc80-mediated inhibition of miR-6937-5p slows down the visual deterioration of rd10 mice, as indicated by the significant greater amplitude in the b-mixed wave under scotopic conditions (Figure 2). This difference in the ERG response was observed both at P17 and P25. In contrast, the rest of the ERG parameters analyzed did not show significant differences after miR-6937-5p inhibition. One possible explanation for this result is that the beneficial effect caused by inhibition of miR-6937-5p derives from the preservation of functional synapses between photoreceptor cells and post-synaptic cells—bipolar cells—thus increasing the b-mixed response. Although no significant differences were detected in the b scotopic response, we observed a tendency towards an increased response at P17 (*p* = 0.14). The difference observed in the effect of miR-6937-5p modulation on b scotopic and b mixed waves could be due to differences in the light intensity exerted and, consequently, the number of rods responding to it. The limited number of rods that respond in the b-scotopic wave register may not be sufficient to allow the detection of significant differences, while in the b-mixed wave, the stimulation of a greater number of cells (rods and cones) would allow the effect of the treatment to be detected.

As for the eyes treated with miR-142a-5p and miR-6240 modulators, no significant differences were observed in any of the parameters analyzed (Figure S4). However, we must take into account that the negative results obtained after the treatment with ssAAV-Anc80-precursor-miR-142a-5p in both the ERGs and the histological studies were likely due, at least in part, to the low rate of infection of the photoreceptor cells yielded with this viral vector. Explanations for this low yield may be related to differences in the design of inhibitory plasmids (used for inhibiting miR6937-5p and miR6240) and miRNA precursors (used for increasing the expression of miR-142a-5p): size, number, and location of promoters or type of vector, among others. Therefore, we cannot raise any conclusion regarding the effects of modulating miR-142a-5p on photoreceptor cell survival in this study.

Motivated by the beneficial effect of miR-6937-5p inhibition on photoreceptor survival and increased ERG response in rd10 mice, we aimed at identifying those genes and biological pathways likely involved in this effect. For this purpose, we performed sub-retinal injections to a new set of rd10 mice at P10. The whole retinal transcriptome was analyzed at P22, after 12 days of miR-6937-5p inhibition using Clariom S arrays (Affymetrix). In addition to retinas from mice treated with AAVs miR-6937-5p inhibitors and AAVs scramble inhibitors (control of miR-effect), we included an additional control group, consisting of mice that received the introduction of the needle without the administration of any treatment of vehicle (control of injection effect). By following this approach, we sought to identify those mRNAs altered exclusively by the effect of miRNA modulation, discarding those mRNAs with differential alteration due to the effects of SR injection or a possible immune response caused by exposure to AAV solution. We identified a total of 101 differentially expressed mRNAs (see Table 1). Among those, we focused on over-expressed genes with possible involvement in the functional improvement of photoreceptors, such as genes related to anti-apoptotic processes (Bcl2a1d, Mapk1, Itch, Arel1 260–263), immune system regulators (Angptl1, Trim21 264–266), inflammatory processes (Jak3 267), and those involved in physiological processes of the retina (Cryba1, Gucy2f, Crygc 204,268,269). Other relevant differentially expressed mRNAs considered included those related to homeostasis or calcium regulation (Atp2c1, Cpne8, Cldn5, Ramp3), transcription regulation (Atxn2l, Tle2, Rps6ka5), or intracellular transport (Klc1, Slc6a9, Cog2), among others.

Nevertheless, among 101 differentially expressed mRNAs, we also found mRNAs with an increased expression reported to negatively affect cell survival, such as the Jmy gene, a cofactor of nuclear p53/TP53 with pro-apoptotic activity, increasing transcription and p53/TP53 dependent apoptosis [47]. Another example is Tm2d1, which has been reported to participate in beta-amyloid-induced apoptosis through its interaction with beta-APP42 [48,49]. Surprisingly, only 3 out of the 101 differentially expressed mRNAs, Angptl1, Bcl2a1d, and Exosc6, are predicted mRNA targets for miR-6937-5p. This indicates that the computational algorithms might be generating many false negatives. Alternatively, there may be compensatory molecular mechanisms that somehow reverse the regulation exercised by miR-6937-5p on some mRNAs, and thus no significant alterations in their expression are detected.

In any case, these three genes hypothetically regulated by miR-6937-5p are associated with biological pathways relevant to retinal degeneration. Therefore, they may be playing an essential role in the mechanisms by which the regulation of miRNA expression generates an improvement in the visual capacity we observed among our rd10 mice.

Angptl1 is associated with the Akt signaling pathway, promoting cell survival and growth in response to extracellular stimuli [50]. Besides, Angptl1 reportedly acts on the negative regulation of cytokine secretion involved in the immune response by inhibiting lipopolysaccharide-induced activation of macrophage cells [51]. However, this gene has also been associated with promoting apoptosis by inhibiting the anti-apoptotic STAT3/Bcl-2 pathway [52]. Therefore, we cannot confirm that the overexpression of Angptl1 improves cell survival in the retina of our mouse model.

Bcl2a1d and Exosc6 are associated with negative regulation of apoptotic processes. Bcl2a1d gene encodes a member of the BCL-2 family of proteins, which act as both anti-apoptotic and pro-apoptotic cellular regulators. In the case of the A1 subfamily of proteins, it has been described that they delay apoptosis by depriving IL-3 [53]. Exosc6 gene encodes for a protein that constitutes one of the subunits of the exosomes, which mediate mRNA degradation. According to the WikiPathways database, this gene is associated with the negative regulation of the apoptotic process of neurons. Regarding the over-expression we observed among the remaining 98 mRNAs (not predicted targets for miR-6937-5p), this may be explained by the existence of indirect gene regulation mechanisms, which might cause a subtle alteration in the expression of intermediary genes, leading to a subsequent significant increase in the expression of secondary mRNAs.

Concerning the in vitro study, we observed increased cytotoxicity in MU-PH1 cells, associated with a 250-fold increased miR-6937-5p expression, supporting a detrimental effect of miR-6937-5p over-expression on a cell line with photoreceptor characteristics.

## 5. Conclusions

In summary, we have found different mRNAs and biological pathways likely involved in the partial functional and structural rescue of photoreceptor degeneration in rd10 mice after retinal inhibition of miR-6937-5p. This work contributes to broadening our knowledge of the molecular mechanisms underlying the establishment and development of retinitis pigmentosa. Interestingly, we succeeded in slowing down the process of photoreceptor and vision loss in a well-established murine model of retinitis pigmentosa by inhibiting miR-6937-5p. These results could lay the foundation for developing therapeutic strategies based on microRNA expression modulation in the future.

## Figures and Tables

**Table 1:** Titration of AAV vectors employed.

ssAAV-Anc-CmiR0001 (scramble precursor)	2.35 × 10^12^ p/mL
ssAAV-Anc-MmiR3437 (precursor miR-142a-5p)	6.96 × 10^11^ vp/mL
ssAAV-Anc-CmiRAN0001 (scramble inhibitor)	9.41 × 10^11^ vp/mL
ssAAV-Anc-MmiRAN2804 (miR-6240 inhibitor)	7.90 × 10^11^ vp/mL
ssAAV-Anc-MmiRAN3970 (inhibitor miR-6937-5p)	1.11× 10^12^ vp/mL

**Table 2:** Expression levels of those differentially expressed mRNAs after in vivo miR-6937-5p modulation and their associated biological pathways. A sample of 10 differentially expressed mRNAs is shown. For a complete list of 101 mRNAs, see Table S2. Abbreviations: SC: scramble miR; C: control (sham injection). (*) indicates predicted target mRNAs for miR-6937-5p.

Gene Symbol	Fold-Change			Pathways
	6937 vs. SC	6937 vs. C	SC vs. C	
* Angptl1	1.5	1.67	−1.12	Akt signaling/Transmembrane receptor protein tyrosine kinase signaling pathway
* Bcl2a1d	1.6	1.5	1.22	Negative regulation of apoptotic process/T cell receptor signaling pathway
* Exosc6	1.62	1.58	1.02	Negative regulation of neuron apoptotic process/rRNA processing
Ntng2	2.31	1.69	1.36	Nervous system development/axonogenesis/cell differentiation
Cpne8	2.25	1.78	1.26	Calcium-dependent membrane-binding
Cryba1	2.16	2.14	1.01	Visual perception/camera-type eye development/lens development in camera-type eye
1600002K03Rik	2.1	1.73	1.22	---
Atxn2l	2.06	1.78	1.16	Regulation of cytoplasmic mRNA processing body assembly/stress granule assembly
Cldn5	2.03	2.39	−1.18	Single organismal cell-cell adhesion/myelination/calcium-independent cell-cell adhesion
Cog2	2.01	1.78	1.13	Protein transport // intra-Golgi vesicle-mediated transport
Mapk1	2	1.65	1.21	Apoptotic process/cellular response to DNA damage stimulus

## Supplementary Materials

The following are available online at https://www.mdpi.com/1999-4923/12/10/913/s1, Figure S1: Maps of the constructs employed for miRNA modulation; Figure S2: Levels of expression of a selected group of miRNAs within rd10 mice retina; Figure S3: Percentage of mice retinal area transduced with AAV vectors after subretinal injection; Figure S4: ERG analysis of rd10 mice submitted to subretinal injections of miRNA modulators with AAVs expressing miR-142a-5p precursors (**A**,**B**) and miR-6240 inhibitors (**C**,**D**); Figure S5: Cytotoxicity of MU-PH1 cell line treated with miR-6937-5p mimics; Table S1: List of validated mRNAs analyzed by qPCR with primer sequences; Table S2: Expression levels of those differentially expressed mRNAs after in vivo miR-6937-5p modulation and their associated biological pathways. Abbreviations: SC: scramble miR; C: control (sham injection). (*) indicates predicted target mRNAs for miR-6937-5p.

## References

[1] RetNet: Summaries. https://sph.uth.edu/retnet/sum-dis.htm.

[2] Donato L., Scimone C., Rinaldi C., Aragona P., Briuglia S., D’Ascola A., D’Angelo R., Sidoti A. (2018). Stargardt phenotype associated with two ELOVL4 promoter variants and ELOVL4 downregulation: New possible perspective to etiopathogenesis?. Investig. Ophthalmol. Vis. Sci..

[3] D’Angelo R., Donato L., Venza I., Scimone C., Aragona P., Sidoti A. (2017). Possible protective role of the ABCA4 gene c.1268A>G missense variant in Stargardt disease and syndromic retinitis pigmentosa in a Sicilian family: Preliminary data. Int. J. Mol. Med..

[4] Scimone C., Donato L., Esposito T., Rinaldi C., D’Angelo R., Sidoti A. (2017). A novel RLBP1 gene geographical area-related mutation present in a young patient with retinitis punctata albescens. Hum. Genom..

[5] Le Meur G., Lebranchu P., Billaud F., Adjali O., Schmitt S., Bézieau S., Péréon Y., Valabregue R., Ivan C., Darmon C. (2018). Safety and Long-Term Efficacy of AAV4 Gene Therapy in Patients with RPE65 Leber Congenital Amaurosis. Mol. Ther..

[6] Wang X., Yu C., Tzekov R.T., Zhu Y., Li W. (2020). The effect of human gene therapy for RPE65-associated Leber’s congenital amaurosis on visual function: A systematic review and meta-analysis. Orphanet J. Rare Dis..

[7] Mellis D., Caporali A. (2018). MicroRNA-based therapeutics in cardiovascular disease: Screening and delivery to the target. Biochem. Soc. Trans..

[8] Kjaer-Frifeldt S., Hansen T.F., Nielsen B.S., Joergensen S., Lindebjerg J., Soerensen F.B., Depont Christensen R., Jakobsen A. (2012). The prognostic importance of miR-21 in stage II colon cancer: A population-based study. Br. J. Cancer.

[9] Loginov V.I., Rykov S.V., Fridman M.V., Braga E.A. (2015). Methylation of miRNA genes and oncogenesis. Biochem..

[10] Bertoli G., Cava C., Castiglioni I. (2016). MicroRNAs as Biomarkers for Diagnosis, Prognosis and Theranostics in Prostate Cancer. Int. J. Mol. Sci..

[11] Cui Z., Zheng X., Kong D. (2016). Decreased miR-198 expression and its prognostic significance in human gastric cancer. World J. Surg. Oncol..

[12] Bhattacharyya S., Balakathiresan N.S., Dalgard C., Gutti U., Armistead D., Jozwik C., Srivastava M., Pollard H.B., Biswas R. (2011). Elevated miR-155 promotes inflammation in cystic fibrosis by driving hyperexpression of interleukin-8. J. Biol. Chem..

[13] Donato L., Scimone C., Alibrandi S., Rinaldi C., Sidoti A., D’angelo R. (2020). Transcriptome analyses of lncrnas in A2E-stressed retinal epithelial cells unveil advanced links between metabolic impairments related to oxidative stress and retinitis pigmentosa. Antioxidants.

[14] Ghanbari M., Darweesh S.K.L., de Looper H.W.J., van Luijn M.M., Hofman A., Ikram M.A., Franco O.H., Erkeland S.J., Dehghan A. (2016). Genetic Variants in MicroRNAs and Their Binding Sites Are Associated with the Risk of Parkinson Disease. Hum. Mutat..

[15] Kumar P., Bhattacharyya S., Peters K.W., Glover M.L., Sen A., Cox R.T., Kundu S., Caohuy H., Frizzell R.A., Pollard H.B. (2015). MIR-16 rescues F508del-CFTR function in native cystic fibrosis epithelial cells. Gene Ther..

[16] Kubota N., Taniguchi F., Nyuya A., Umeda Y., Mori Y., Fujiwara T., Tanioka H., Tsuruta A., Yamaguchi Y., Nagasaka T. (2020). Upregulation of microRNA-31 is associated with poor prognosis in patients with advanced colorectal cancer. Oncol. Lett..

[17] Luly F.R., Lévêque M., Licursi V., Cimino G., Martin-Chouly C., Théret N., Negri R., Cavinato L., Ascenzioni F., Del Porto P. (2019). MiR-146a is over-expressed and controls IL-6 production in cystic fibrosis macrophages. Sci. Rep..

[18] Muñoz-Culla M., Irizar H., Sáenz-Cuesta M., Castillo-Triviño T., Osorio-Querejeta I., Sepúlveda L., López De Munain A., Olascoaga J., Otaegui D. (2016). SncRNA (microRNA &snoRNA) opposite expression pattern found in multiple sclerosis relapse and remission is sex dependent. Sci. Rep..

[19] van Rooij E., Marshall W.S., Olson E.N. (2008). Toward microRNA-based therapeutics for heart disease: The sense in antisense. Circ. Res..

[20] Xu P., Zhu Y., Sun B., Xiao Z. (2016). Colorectal cancer characterization and therapeutic target prediction based on microRNA expression profile. Sci. Rep..

[21] Sun L., Chen X., Jin Z. (2020). Emerging roles of noncoding RNAs in retinal diseases: A review. Clin. Exp. Ophthalmol..

[22] Mortuza R., Feng B., Chakrabarti S. (2014). MiR-195 regulates SIRT1-mediated changes in diabetic retinopathy. Diabetologia.

[23] Kaidonis G., Gillies M.C., Abhary S., Liu E., Essex R.W., Chang J.H., Pal B., Sivaprasad S., Pefkianaki M., Daniell M. (2016). A single-nucleotide polymorphism in the MicroRNA-146a gene is associated with diabetic nephropathy and sight-threatening diabetic retinopathy in Caucasian patients. Acta Diabetol..

[24] Lukiw W.J., Surjyadipta B., Dua P., Alexandrov P.N. (2012). Common micro RNAs (miRNAs) target complement factor H (CFH) regulation in Alzheimer’s disease (AD) and in age-related macular degeneration (AMD). Int. J. Biochem. Mol. Biol..

[25] Bhattacharjee S., Zhao Y., Dua P., Rogaev E.I., Lukiw W.J. (2016). MicroRNA-34α-mediated down-regulation of the microglial-enriched triggering receptor and phagocytosis-sensor TREM2 in age-related macular degeneration. PLoS ONE.

[26] Bai S., Tian B., Li A., Yao Q., Zhang G., Li F. (2016). MicroRNA-125b promotes tumor growth and suppresses apoptosis by targeting DRAM2 in retinoblastoma. Eye.

[27] Loscher C.J., Hokamp K., Wilson J.H., Li T., Humphries P., Farrar G.J., Palfi A. (2008). A common microRNA signature in mouse models of retinal degeneration. Exp. Eye Res..

[28] Loscher C.J., Hokamp K., Kenna P.F., Ivens A.C., Humphries P., Palfi A., Farrar G.J. (2007). Altered retinal microRNA expression profile in a mouse model of retinitis pigmentosa. Genome Biol..

[29] Donato L., Bramanti P., Scimone C., Rinaldi C., D’Angelo R., Sidoti A. (2018). miRNA expression profile of retinal pigment epithelial cells under oxidative stress conditions. FEBS Open Bio.

[30] Anasagasti A., Ezquerra-Inchausti M., Barandika O., Culla M.M., Caffarel M.M., Otaegui D., López de Munain A., Ruiz-Ederra J. (2018). Expression profiling analysis reveals key Microrna-mRNA interactions in early retinal degeneration in retinitis pigmentosa. Investig. Ophthalmol. Vis. Sci..

[31] Ban E., Kwon T.-H., Kim A. (2019). Delivery of therapeutic miRNA using polymer-based formulation. Drug Deliv. Transl. Res..

[32] Ye Y., Li Z., Feng Q., Chen Z., Wu Z., Wang J., Ye X., Zhang D., Liu L., Gao W. (2017). Downregulation of microRNA-145 may contribute to liver fibrosis in biliary atresia by targeting ADD3. PLoS ONE.

[33] Ji H.P., Xiong Y., Song W.T., Zhang E.D., Gao Z.L., Yao F., Su T., Zhou R.R., Xia X.B. (2017). MicroRNA-28 potentially regulates the photoreceptor lineage commitment of Müller glia-derived progenitors. Sci. Rep..

[34] Fernando N., Wong J.H.C., Das S., Dietrich C., Aggio-Bruce R., Cioanca A.V., Wooff Y., Chu-Tan J.A., Schumann U., Ngo C. (2020). MicroRNA-223 Regulates Retinal Function and Inflammation in the Healthy and Degenerating Retina. Front. Cell Dev. Biol..

[35] Murillo O., Luqui D.M., Gazquez C., Martinez-Espartosa D., Navarro-Blasco I., Monreal J.I., Guembe L., Moreno-Cermeño A., Corrales F.J., Prieto J. (2016). Long-term metabolic correction of Wilson’s disease in a murine model by gene therapy. J. Hepatol..

[36] Corrochano S., Barhoum R., Boya P., Arroba A.I., Rodríguez-Muela N., Gómez-Vicente V., Bosch F., De Pablo F., De La Villa P., De La Rosa E.J. (2008). Attenuation of vision loss and delay in apoptosis of photoreceptors induced by proinsulin in a mouse model of retinitis pigmentosa. Investig. Ophthalmol. Vis. Sci..

[37] Gómez-Vicente V., Flores A., Lax P., Murciano C., Yáñez A., Gil M.L., Cuenca N., Gozalbo D., Maneu V. (2013). Characterization of a new murine retinal cell line (mu-ph1) with glial, progenitor and photoreceptor characteristics. Exp. Eye Res..

[38] Toral-Ojeda I., Aldanondo G., Lasa-Elgarresta J., Lasa-Fernandez H., Vesga-Castro C., Mouly V., de Munain A.L., Vallejo-Illarramendi A. (2018). A Novel Functional In Vitro Model that Recapitulates Human Muscle Disorders. Muscle Cell and Tissue—Current Status of Research Field.

[39] Peng B., Xiao J., Wang K., So K.-F., Tipoe G.L., Lin B. (2014). Suppression of microglial activation is neuroprotective in a mouse model of human retinitis pigmentosa. J. Neurosci..

[40] Lu T.X., Rothenberg M.E. (2018). MicroRNA. J. Allergy Clin. Immunol..

[41] Rupaimoole R., Slack F.J. (2017). MicroRNA therapeutics: Towards a new era for the management of cancer and other diseases. Nat. Rev. Drug Discov..

[42] Donato L., D’Angelo R., Alibrandi S., Rinaldi C., Sidoti A., Scimone C. (2020). Effects of A2E-induced oxidative stress on retinal epithelial cells: New insights on differential gene response and retinal dystrophies. Antioxidants.

[43] Donato L., Scimone C., Alibrandi S., Nicocia G., Rinaldi C., Sidoti A., D’Angelo R. (2020). Discovery of glo1 new related genes and pathways by rna-seq on a2e-stressed retinal epithelial cells could improve knowledge on retinitis pigmentosa. Antioxidants.

[44] Palfi A., Hokamp K., Hauck S.M., Vencken S., Millington-Ward S., Chadderton N., Carrigan M., Kortvely E., Greene C.M., Kenna P.F. (2016). MicroRNA regulatory circuits in a mouse model of inherited retinal degeneration. Sci. Rep..

[45] Zhao Y., Fent K. (2016). Progestins alter photo-transduction cascade and circadian rhythm network in eyes of zebrafish (Danio rerio). Sci. Rep..

[46] Zinn E., Pacouret S., Khaychuk V., Turunen H.T., Carvalho L.S., Andres-Mateos E., Shah S., Shelke R., Maurer A.C., Maurer E. (2015). In silico reconstruction of the viral evolutionary lineage yields a potent gene therapy vector. Cell Rep..

[47] Adighibe O., Pezzella F. (2018). The Role of JMY in p53 Regulation. Cancers.

[48] Kajkowski E.M., Lo C.F., Ning X., Walker S., Sofia H.J., Wang W., Edris W., Chanda P., Wagner E., Vile S. (2001). β-Amyloid Peptide-induced Apoptosis Regulated by a Novel Protein Containing a G Protein Activation Module. J. Biol. Chem..

[49] Lee Y., Chang D.J., Lee Y.S., Chang K.A., Kim H., Yoon J.S., Lee S., Suh Y.H., Kaang B.K. (2003). β-amyloid peptide binding protein does not couple to G protein in a heterologous Xenopus expression system. J. Neurosci. Res..

[50] Chen H.A., Kuo T.C., Tseng C.F., Ma J.T., Yang S.T., Yen C.J., Yang C.Y., Sung S.Y., Su J.L. (2016). Angiopoietin-like protein 1 antagonizes MET receptor activity to repress sorafenib resistance and cancer stemness in hepatocellular carcinoma. Hepatology.

[51] Gu H., Cui M., Bai Y., Chen F., Ma K., Zhou C., Guo L. (2010). Angiopoietin-1/Tie2 signaling pathway inhibits lipopolysaccharide-induced activation of RAW264.7 macrophage cells. Biochem. Biophys. Res. Commun..

[52] Yan Q., Jiang L., Liu M., Yu D., Zhang Y., Li Y., Fang S., Li Y., Zhu Y.H., Yuan Y.F. (2017). ANGPTL1 interacts with integrin α1β1 to suppress HCC angiogenesis and metastasis by inhibiting JAK2/STAT3 signaling. Cancer Res..

[53] Cartagena C.M., Schmid K.E., Phillips K.L., Tortella F.C., Dave J.R. (2013). Changes in apoptotic mechanisms following penetrating ballistic-like brain injury. J. Mol. Neurosci..

