# Supplementary material for: Inhibition of MicroRNA 6937 Delays Photoreceptor and Vision Loss in a Mouse Model of Retinitis Pigmentosa
Source: Pharmaceutics. 2020 Sep 24;12(10):913. doi: 10.3390/pharmaceutics12100913 (PMC7598722; doi:10.3390/pharmaceutics12100913)
Supplement: Supplementary file 1 [file pharmaceutics-12-00913-s001.pdf]

Supplementary Materials: Inhibition of MicroRNA 6937 Delays Photoreceptor and Vision Loss in a Mouse Model of Retinitis Pigmentosa

Ander Anasagasti, Araceli Lara-López, Santiago Milla-Navarro, Leire Escudero-Arrarás, María Rodríguez-Hidalgo, Nerea Zabaleta, Gloria González Aseguinolaza, Pedro de la Villa, Javier Ruiz-Ederra.

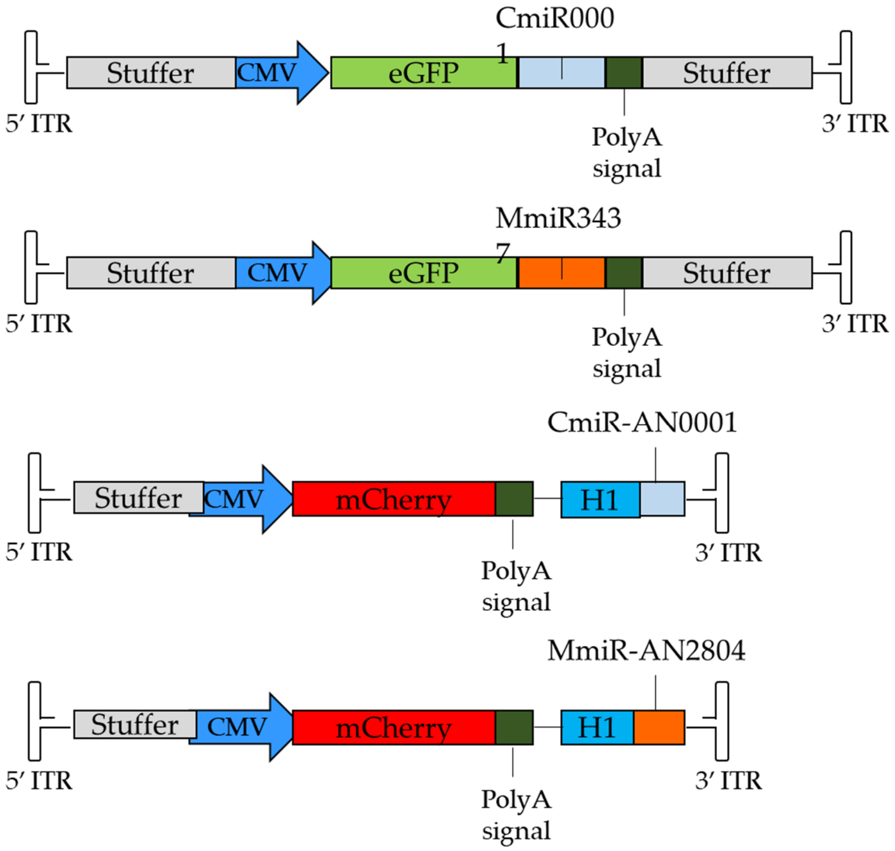

Figure S1. Maps of the constructs employed for miRNA modulation**.** Pre-miRNAs expressed green fluorescence reporter (eGFP, top), whereas anti-miRNA expressed red fluorescence reporter (mCherry), under the same promoter (CMV). Constructs of scramble sequences for both pre- and anti-miRNAs (controls) is also shown.

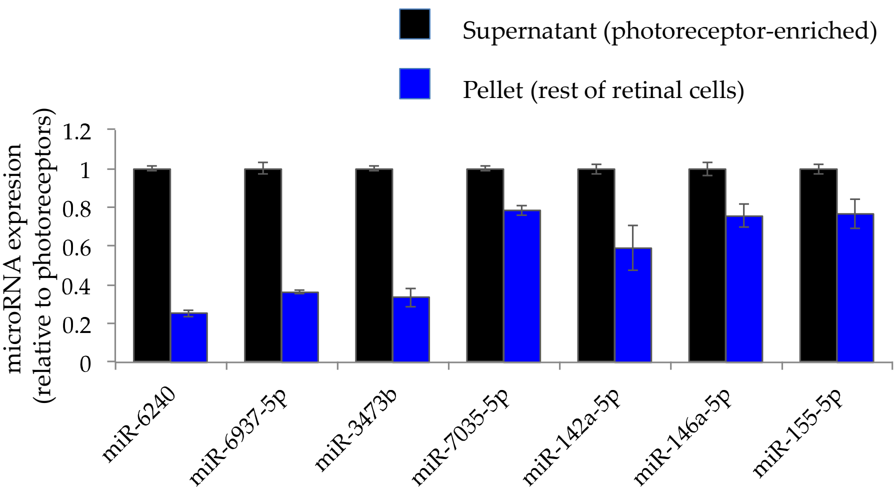

Figure S2. Levels of expression of a selected group of microRNAs within rd10 mice retina. Expression levels of non-photoreceptor retinal cells, in blue, are normalized to isolated photoreceptors, in black. Data is represented as mean values +/- SEM.

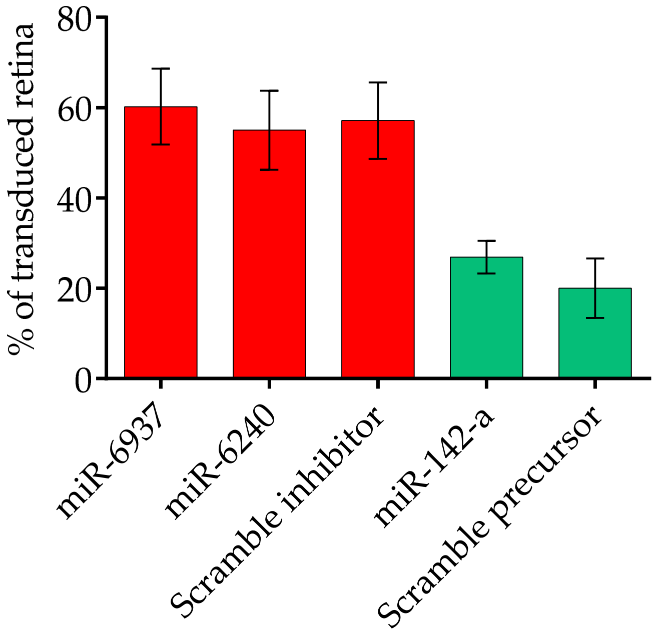

Figure S3. Percentage of mice retinal area transduced with AAV vectors after subretinal injection. AAVs expressing inhibitory miR sequences included mCherry reporter, and are represented as red bars. We were able to transfect up to 60% of retinal area. AAVs expressing precursor miR sequences included GFP reporter, and are represented as green bars. Only 20–30% of retinal area was successfully transfected using miR precursors. Data is represented as mean values +/- SEM.

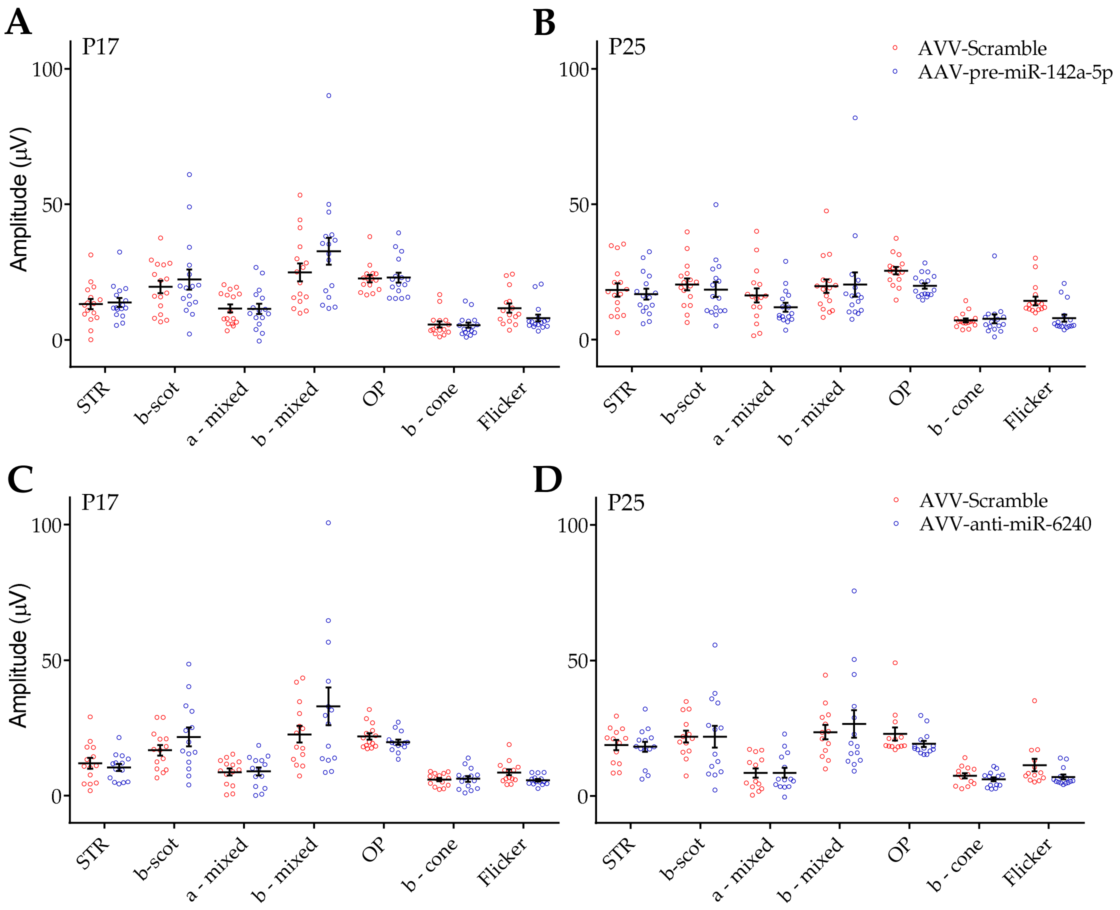

Figure S4. Representative histograms of different ERG parameters analyzed after subretinal injection with AAVs expressing miR-142a-5p precursors (A-B) and miR-6240 inhibitors (C-D). No differences with respect controls were observed in any parameter analyzed neither at P17 nor at P22. *N* = 15 mice per treatment. Abbreviations: STR: Scotopic threshold response; scot: scotopic; OP: Oscillatory Potentials. . Data is represented as mean values +/- SEM.

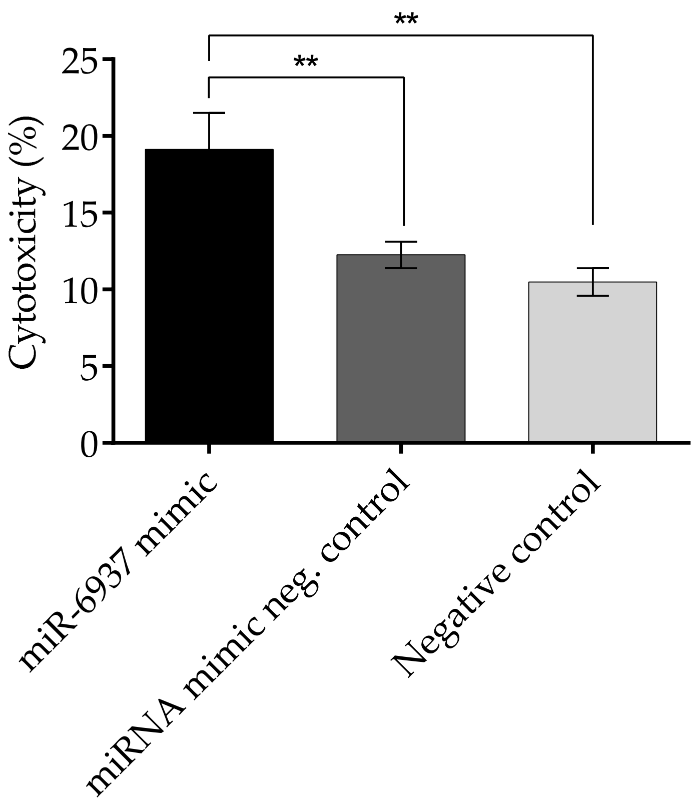

Figure S5. Cytotoxicity of MU-PH1 cell line treated with miR-6937-5p mimics. An increased citotoxicity is associated with miR-6937-5p expression. *N* = 6 plates/condition in 3 independent experiments. Data is represented as mean values +/- SEM (**p < 0.005, student's t-test).

Table S1. List of validated mRNAs analyzed by qPCR with primer sequences.

mRNA ID	Forward Primer	Reverse Primer
Arhgap18	AGACAACTTCGTTTTCGGTCAGTCT	ACCAACTCAGCTCCCTCTGTGTAGT
Cngb1	CTGGAGAGGCATGCAGAAGGT	GGATCTGTGCTCCACCCTGAT
Crx	TCCAGGGTCCAGGTCTGGTT	CTGTTGCTGTTTCTGCTGCTGT
Cryaa	GCCCTTCCTGTCTTCCACCAT	CCACATATGGGTCATGAGCTCAGA
Cth	GCTTCGTTTCCTGCAGAATTCACTA	CATCCGGACCTGCAGTGTCTT
Cxcr4	GCCATGGAACCGATCAGTGTGAGTATA	ACGTTTTCATCCCGGAAGCA
Doc2b	GGACCACAATGGACTGGCTGAT	TTGTTCTGAGCTTATTTGCCTTGCT
Dpys15	GAAGCTGGTACAGAGAGAGAAGACCTTAA	GGTGGACAACAATGGCGACAT
Duxbl2	CTCTCGAGTCTCAAATTATGACTTGGTTT	TCTAGGTTTATCCTGCTCCTGCTCTT
E2f3	CCAAGCCCACTTCCAAAGACTT	GGATTTGGTCCTCAGTCTGCTGTA
Eif2s3y	CAGGTGGTCTTATTGGAGTTGGA	TCTAACTCTGTGAATATCTCAGGTAATGCT
Fabp7	CACAATGCACATTCAAGAACACAGA	ATCCAACCGAACCACAGACTTACA
Klhl23	GACCGTAGTTTCGCCTTCTTCTCT	TGTCCTTTTAGAGCCATGCTTCTCT
Lca5	AAGTGCATCACCCAACCGTTA	ATCTTTCCGAAGTGGCTCTCTGT
Map2k6	TGTCTGTTATCCATCGAGACGTCAA	AGAGTCGACCAGGTAGCCACTGAT
Mllt11	TGAGGCATCCCTTCCGTCTT	GGTCCCTCATGATCCCCTGTTA
Mtch1	CTGCTGATCCAGGTGGGTCAT	AGGTGAAGAAGCTCGGCAGGTA
Mtfp1	CAAATGGACCACCACCACACTT	AGGAAGTCCACTGACCTGTCGAT
Nnat	GTGCTGCTGCAGGTGTTCCT	GGCTGTGTCCCTGGAGGATT
Nr2e3	CAGGTGATGCTAAGCCAGCAT	GAGCAATTTCCCAAACCTCACA
Nxnl2	GACCCCTACCGGCATGAACT	TGACAGCTCCGTTCTGCTTGA
Osmr	CCAGACACAAAGTACAAGGCTTTTGT	AGAGCCTGTGAGGGAGCAGTCT
Otud7b	CCATGACCCTGGACATGGAT	ACTCACATCCCAGTTCTTTCCTTCT
Pak3	TGCTAAGGAGCTTTTGCAGCAT	CGGCTACTGTTCTTAATTGCTTCCT
Pan2	GCTATCATCTCCCAGTCAGGTCAAT	CACATCAAACGTCATTAGCAAAGGT
Pde6g	AGCGGCAAACAAGGCAGTT	TGCAAGGGTTTGGGGATGA
Peli3	CCAGGTGTCTGGAGGGAGATT	GGATTCGTTTTCCACCAGCTT
Pla2r1	CCACCACAAGCCGATACGAA	TCACAGAACACCTTCATAGAGGTAGGAT
Prdm1	CTGAAGAAACAGAATGGCAAGATCAA	TGCACTCTCAGGTGGACCTTCA
Psd2	GAGTTCAGCAGGCTGGTTGCT	GGCCTTCAGGAAGGTTCTGAGT
Ptn	CAGACCATGAAAGACTCAGAGATGTAAGAT	ACTGGTACTTGCACTCAGCTCCAA
Rdh12	ACAGCTCCATCCATCAGGAAGTT	TCTGGCTGTCTCCTTGCCAAT
Rdh8	GGACAAGTGGATGTGCTAGTGAACA	TGCATGGTGGCTAGGCTGAGT
Rgr	TGACTATGAGCCTGTGGGGACAT	TGAAGAGAAAACTGATGAAGTTTCTGTCA
Rraga	CATCTGGGATGAGACGCTCTACA	CTGGGCAAAATTCCTTAGGTTCA
Rs1	GATCTTGCTGTGACCAAGGACAA	GACAATCCCAATGTGGCTTCA
Sema7a	CCATCCGGAAGCAGGAATACA	TTGATGAACTGTGGGTTCTGCAT
Serpina3n	CTGGAGGATGTCCTTTCAAAGCT	GCCTTGTGGACCACCTGAGA
Sgk1	GACTACATTAATGGTGGAGAGCTGTTCTA	GCTATTTCAGCTGCGTAGAATCGA
Tdrd7	ACCGTCTGTGTTGGTGGTTGA	AGCTGAGTAGTCTTTGCCTACATACCTGATA

Table S2. Expression levels of those differentially expressed mRNAs after in vivo miR-6937-5p modulation and their associated biological pathways. Abbreviations: SC: scramble miR; C: control (sham injection). (*) indicates predicted target mRNAs for miRA6937A5p.

Gene Symbol	Fold-change			Pathways
	937 vs SC	6937 vs C	SC vs. C	
*** Angptl1**	1.5	1.67	−1.12	Akt signaling / Transmembrane receptor protein tyrosine kinase signaling pathway
* Bcl2a1d	1.6	1.5	1.22	Negative regulation of apoptotic process / T cell receptor signaling pathway
* Exosc6	1.62	1.58	1.02	Negative regulation of neuron apoptotic process / rRNA processing
Ntng2	2.31	1.69	1.36	Nervous system development / axonogenesis / cell differentiation
Cpne8	2.25	1.78	1.26	Calcium-dependent membrane-binding
Cryba1	2.16	2.14	1.01	Visual perception / camera-type eye development / lens development in camera-type eye
1600002K03Rik	2.1	1.73	1.22	---
Atxn2l	2.06	1.78	1.16	Regulation of cytoplasmic mRNA processing body assembly / stress granule assembly
Cldn5	2.03	2.39	−1.18	Single organismal cell-cell adhesion / myelination / calcium-independent cell-cell adhesion
Cog2	2.01	1.78	1.13	Protein transport // intra-Golgi vesicle-mediated transport
Mapk1	2	1.65	1.21	Apoptotic process / cellular response to DNA damage stimulus
Lmln	1.98	1.57	1.26	Proteolysis / cell cycle / mitotic nuclear division
Tle2	1.93	1.68	1.15	Regulation of transcription. DNA-templated / Wnt signaling pathway
Fancc	1.92	1.59	1.2	Myeloid cell homeostasis / DNA repair / cellular response to DNA damage stimulus
Ipo9	1.91	1.51	1.26	Protein import into nucleus / ribosomal protein import into nucleus / transport
Hnrnph3	1.9	1.54	1.24	Nucleic acid binding / RNA binding
Tmem242	1.9	1.71	1.11	Transmembrane Protein
Defb110	1.89	1.8	1.05	Innate Immune System / Defense response
Defb41	1.89	1.8	1.05	---
Csnk1g2	1.87	1.71	1.1	Protein phosphorylation / Wnt signaling pathway
Gm2027	1.87	1.52	1.23	---
Pnpla8	1.87	1.52	1.23	Cell death / lipid metabolic process / fatty acid metabolic process
Tmem182a	1.85	1.5	1.23	Toxic substance binding / pathogenesis
Blvra	1.83	1.69	1.08	Heme catabolic process / oxidation-reduction process
Slc6a9	1.83	1.75	1.04	Neurotransmitter transport / amino acid transport / glycine transport /
Clip1	1.81	1.66	1.09	Transport / positive regulation of microtubule polymerization / microtubule bundle formation
Ipo13	1.79	2.18	−1.22	Protein import into nucleus / transport / intracellular protein transport
Dot1l	1.79	1.68	1.07	Chromatin modification / histone methylation / peptidyl-lysine methylation
Ckmt2	1.77	1.55	1.14	Phosphocreatine metabolic process / phosphorylation
Spc25	1.76	1.91	−1.08	Cell cycle / chromosome segregation / mitotic nuclear division
Tma16	1.76	1.6	1.1	Biological process
Heatr5a	1.76	1.78	−1.01	Binding
Sars	1.75	1.88	−1.08	Translation / tRNA aminoacylation for protein translation / seryl-tRNA aminoacylation

Slc7a8	1.73	1.72	1	Amino acid transmembrane transport / peptide antigen binding
Slc7a5	1.71	1.5	1.14	Amino acid transmembrane transport / transport
Nt5c2	1.71	1.52	1.12	IMP metabolic process / adenosine metabolic process
Scfd1	1.7	1.71	−1.01	Retrograde vesicle-mediated transport. Golgi to ER / vesicle docking involved in exocytosis
Syf2	1.67	2.01	−1.21	mRNA processing / mitotic G2 DNA damage checkpoint / RNA splicing
Frmd4b	1.67	1.86	−1.11	Establishment of epithelial cell polarity
Jmy	1.67	2.37	−1.42	Regulation of apoptotic process / intrinsic apoptotic signaling pathway by p53 class mediator
Crygc	1.66	1.5	1.18	Eye development / visual perception / camera-type eye development
Rps6ka5	1.66	1.96	−1.18	Regulation of transcription. DNA-templated
G6pc3	1.65	1.57	1.05	Gluconeogenesis / phosphate-containing compound metabolic process
Slc6a8	1.64	1.61	1.02	Transport / ion transport / sodium ion transport / neurotransmitter transport
Mettl16	1.64	1.95	−1.18	Methylation
U2af1	1.64	1.6	1.03	mRNA processing / RNA splicing
Tspan31	1.63	2.09	−1.29	Regulation of cell development. Activation. growth and motility
Tmem181b-ps	1.63	1.82	−1.12	pathogenesis
Tmem181a	1.63	1.82	−1.12	pathogenesis
Aars	1.62	1.58	1.02	tRNA Aminoacylation
Atp2c1	1.62	1.86	−1.14	Cellular calcium ion homeostasis / signal transduction / ATP catabolic process / transport
Ankrd60	1.61	1.5	1.08	---
Pfdn2	1.59	1.58	1	Protein folding
Tm4sf1	1.59	1.84	−1.16	---
Slc25a44	1.59	1.78	−1.12	Transport / transmembrane transport
Afg3l1	1.59	1.57	1.01	Proteolysis / mitochondrial protein processing
Gucy2f	1.59	1.95	−1.22	Visual perception / cGMP biosynthetic process / phototransduction
R3hdm4	1.59	1.53	1.04	nucleic acid binding
Dcaf12	1.58	1.83	−1.15	Protein ubiquitination
Ireb2	1.58	1.79	−1.14	Regulation of translation / cellular iron ion homeostasis
Gm10477	1.58	1.77	−1.12	---
Thg1l	1.58	1.67	−1.06	tRNA modification / tRNA processing / protein homotetramerization
Rhot2	1.58	1.88	−1.19	cellular homeostasis / mitochondrion transport along microtubule
Gm16503	1.57	1.53	1.02	---
Enkd1	1.57	1.66	−1.06	---
Tm2d1	1.57	1.65	−1.06	Apoptotic process / G-protein coupled receptor signaling pathway
Ramp3	1.57	1.5	1.05	Calcium ion transport / intracellular protein transport / signal transduction
Arel1	1.57	1.69	−1.08	Negative regulation of apoptotic process / Protein ubiquitination
Gm12689	1.56	1.62	−1.04	---
Sval1	1.55	1.54	1.01	---
Sae1	1.55	1.62	−1.05	Cellular protein modification process / protein sumoylation / regulation of mitotic cell cycle
Gm14525	1.55	1.66	−1.07	---
Trim21	1.55	1.58	1.12	Regulation of Innate immune response / positive regulation of cell cycle / protein polyubiquitination
Klc1	1.55	1.6	−1.03	Intracellular protein transport / microtubule-based movement
Wdr27	1.55	1.5	1.03	Protein-protein interaction / cell signaling

Kcnd1	1.54	1.74	−1.13	Transport / ion transport / potassium ion transport / regulation of ion transmembrane transport
Mtrf1	1.54	1.61	−1.04	Translational termination / Nucleotide binding and translation release factor activity
Dolk	1.53	1.69	−1.11	Phosphorylation / dolichyl monophosphate biosynthetic process
Rragd	1.53	1.59	−1.04	RET signaling / Translational Control
Abcb1a	1.53	2.04	−1.34	G2/M transition of mitotic cell cycle / ATP catabolic process / drug transmembrane transport
Gpn3	1.53	1.53	1	Nucleotide binding / hydrolase activity
Tfpt	1.53	1.69	−1.1	DNA repair / apoptotic process / DNA recombination / regulation of transcription. DNA-templated
Hnrnpu	1.52	1.7	−1.12	mRNA processing / RNA splicing / osteoblast differentiation
Itch	1.52	1.65	−1.09	Negative regulation of apoptotic process / Immune system process / positive regulation of T cell anergy
Slc30a7	1.52	1.53	−1.01	Transport / transmembrane transport / ion transport / cation transport / zinc ion transport
Slc17a5	1.52	1.61	−1.06	Transport / amino acid transport / sialic acid transport / ion transmembrane transport
Jak3	1.52	1.61	−1.06	Negative regulation of cytokine production / regulation of cytosolic calcium concentration
Dhx16	1.52	1.61	−1.06	ATP catabolic process
Ifi44	1.51	1.95	−1.29	Heart Block. Congenital
Vps13d	1.51	1.5	1.01	Choreoacanthocytosis
Wdr54	1.51	1.58	−1.05	---
Ctdnep1	1.51	1.7	−1.13	Positive regulation of canonical Wnt signaling pathway / protein dephosphorylation
Cd68	1.51	1.9	−1.26	Cellular response to organic substance
Drg1	1.51	1.62	−1.07	Ferrous iron transport
Ak2	1.5	1.65	−1.1	ADP biosynthetic process / phosphorylation / AMP metabolic process
Tceanc2	1.5	2.23	−1.49	Transcription. DNA-templated
Huwe1	1.5	1.61	−1.07	Protein ubiquitination / DNA repair / cellular response to DNA damage stimulus
Xpo1	1.5	2.23	−1.49	Negative regulation of transcription from RNA polymerase II promoter / protein export from nucleus
March6	1.5	1.64	−1.09	Protein K48-linked ubiquitination
Pla2g7	1.5	1.58	−1.05	Regulation of monocyte chemotaxis /inflammatory response/calcium-independent phospholipase activity
Tm9sf3	1.5	1.87	−1.25	Assembly and activity of V-ATPase
